# Effects of acculturative stress on PTSD, depressive, and anxiety symptoms among refugees resettled in Australia and Austria

**DOI:** 10.3402/ejpt.v7.28711

**Published:** 2016-02-12

**Authors:** Dzenana Kartal, Litza Kiropoulos

**Affiliations:** 1 Department of Psychiatry, School of Clinical Sciences, Monash University, Clayton, Australia; 2 Melbourne School of Psychological Sciences, University of Melbourne, Parkville, Australia; 3 Psychology Department, Royal Melbourne Hospital, Parkville, Australia

**Keywords:** Trauma, refugees, acculturative stress, posttraumatic stress disorder (PTSD), depression, anxiety

## Abstract

**Background:**

Research indicates that exposure to war-related traumatic events impacts on the mental health of refugees and leads to higher rates of posttraumatic stress disorder (PTSD), depression, and anxiety symptoms. Furthermore, stress associated with the migration process has also been shown to impact negatively on refugees' mental health, but the extent of these experiences is highly debatable as the relationships between traumatic events, migration, and mental health outcomes are complex and poorly understood.

**Objective:**

This study aimed to examine the influence of trauma-related and post-migratory factors on symptoms of PTSD, depression, and anxiety in two samples of Bosnian refugees that have resettled in two different host nations—Austria and Australia.

**Method:**

Using multiple recruitment methods, 138 participants were recruited to complete self-report measures assessing acculturative stress, PTSD, depressive, and anxiety symptoms.

**Results:**

Hierarchical regressions indicated that after controlling for age, sex, and exposure to traumatic events, acculturative stress associated with post-migratory experiences predicted severity of PTSD and anxiety symptoms, while depressive symptoms were only predicted by exposure to traumatic events. This model, however, was only significant for Bosnian refugees resettled in Austria, as PTSD, depressive, and anxiety symptoms were only predicted by traumatic exposure in the Bosnian refugees resettled in Australia.

**Conclusion:**

These findings point toward the importance of assessing both psychological and social stressors when assessing mental health of refugees. Furthermore, these results draw attention to the influence of the host society on post-migratory adaptation and mental health of refugees. Further research is needed to replicate these findings among other refugee samples in other host nations.

Epidemiological research in the area of posttraumatic mental health indicates that prior trauma is a significant predictor of posttraumatic stress disorder (PTSD) (Ozer, Ozer, Best, Lipsey, & Weiss, 2008), and that depressive and anxiety symptoms are prevalent comorbid outcomes of traumatic experiences in general (Australian Centre for Posttraumatic Mental Health, 2013) and refugee populations (Fazel, Wheeler, & Danesh, 2005; Kirmayer et al., 2011; Steel, Silove, Phan, & Bauman, 2002; Steel et al., 2009). In addition, challenges associated with migration to a new country have been found to increase the risk of mental health problems in refugees (Porter & Haslam, 2005; Steel, Silove, Bird, McGorry, & Mohan, 1999; Steel et al., 2002). These post-migratory challenges are often related to acculturation, defined as the process of simultaneous participation with the new culture and maintenance of the origin culture and identity (Berry, 1997). Acculturation is mutually influenced and changed by attitudes of the individual as much as the attitudes and preferences of the ethnic and host groups (Berry, 2003). The consequences of the process of acculturation have been found to be substantial and to influence mental health outcomes in migratory groups and individuals (Bhugra, 2004; Sam & Berry, 2010).

Research has found inconsistent results indicating positive, negative, or no association between acculturation and mental health outcomes in refugees (Aichberger et al., 2015; Berry, Phinney, Sam, & Vedder, 2006; Bhugra, 2003; Birman & Tyler, 1994; Escobar & Vega, 2000; Li & Anderson, 2015; Mölsä et al., 2014; Schwartz, Schwartz, Unger, Zamboanga, & Szapocznik, 2010; Syed et al., 2006). Furthermore, most empirical evidence has concentrated on exploring the acculturative process of the individual without exploring the impact of the host society, which has been suggested to be important in understanding the full process of acculturation and stress associated with migration (Schwartz et al., 2010). The question therefore remains whether the influence of post-migratory demands on mental health differs based on the individual's acculturation process alone, or is it also dependent on the characteristics of the local context reflecting the acculturative preferences of the host society.

This study examines the relative contribution of pre-migratory traumatic experiences and post-migratory acculturative stress in predicting mental health outcomes in Bosnian refugees who have resettled in two countries and explores the potential role of the local context in the acculturative process.

## Traumatic exposure, acculturation, and mental health outcomes

Research evidence suggests that refugees are exposed to multiple, sometimes extreme traumas such as torture, rape, or death of family members (Steel et al., 1999, 2002), which puts them at higher risk for developing serious mental health problems (Steel et al., 2009). Compared to the general population, refugees can be about five to ten times more likely to present with depression and PTSD symptoms respectively (Fazel et al., 2005). While there is substantial evidence to indicate that trauma exposure is a risk factor for PTSD, depression, and anxiety symptoms (Ozer et al., 2008; Steel et al., 2009), empirical evidence on the relationship between acculturation and mental health is less consistent. Some research suggests that higher levels of acculturation with the host culture are associated with better mental health, while others reported that higher levels of acculturation with the host culture is associated with worse mental health outcomes—a phenomenon named the “immigrant paradox” (for discussion see: Berry et al., 2006; Bhugra, 2003, 2004; Schwartz et al., 2010). Specific acculturative factors however, present a more consistent relationship with mental health. For example, acculturative stress experienced in response to migratory challenges is regularly identified as a significant risk factor for mental health problems (Berry, 2006b; Bogic et al., 2012; Ellis, MacDonald, Lincoln, & Cabral, 2008; Knipscheer & Kleber, 2006), and specifically associated with higher levels of PTSD symptoms (Jorden, Matheson, & Anisman, 2009; Nicholson, 1997; Schweitzer, Brough, Vromans, & Asic-Kobe, 2011), depressive symptoms (Fenta, Hyman, & Noh, 2004; Jorden et al., 2009; Schweitzer et al., 2011), and anxiety symptoms (Schweitzer, Melville, Steel, & Lacherez, 2006; Schweitzer et al., 2011). Specifically, acculturative stressors relating to lower socioeconomic status (Syed et al., 2006), unstable working conditions and unemployment (Beiser & Hou, 2001; Mölsä et al., 2014; Teodorescu, Heir, Hauff, Wentzel-Larsen, & Lien, 2012), lower language acquisition (Söndergaard & Theorell, 2004), and perceived discrimination (Aichberger et al., 2015; Ellis et al., 2010; Li & Anderson, 2015) have been found to contribute to poorer mental health outcomes. These findings have been demonstrated even after accounting for the effect of pre-migratory traumatic exposure, indicating a potential cumulative effect of post-migratory acculturative factors.

## Theoretical framework

Acculturation is defined as a two-dimensional process underlined by the *cultural maintenance* of the culture of origin and *contact and participation* with the host culture (Berry, 1997). Early definitions of acculturation were criticised because they were based on the assumption that non-dominant groups can always choose which acculturation style they want to adapt (Berry, 1974). Unfortunately, this is not always possible and it is now widely accepted that the acculturative experience depends on the conditions of the host society as much as they depend on the individual acculturative preferences (Berry, 1997, 2006a). The variations in the conditions or contexts of acculturation are identified as acculturation strategies and represent the preferred acculturation attitudes of the host societies. The first, *melting pot*, refers to the attitudes of the host society when the assimilation of all the non-dominant groups is sought or even enforced. When separation or marginalisation of the non-dominant groups is sought or enforced by the host society, this is known as *segregation* and *exclusion* respectively. The last, *multiculturalism*, refers to attitudes of the society where cultural diversity is valued and encouraged by the host society as a whole (Berry, 1997, 2006a; Berry et al., 2006). Hence, while it can be argued that the most contemporary societies incorporate groups of various cultural and ethnic backgrounds, they differ in their policies as to how these groups should live together in the larger society and how they maintain their cultural and ethnic distinctiveness.

The current study proposes to consider the influence of acculturative factors by investigating the association between acculturative stress and mental health outcomes of refugees in two different acculturative contexts that differ in the strength of their multicultural policies. In the absence of the classification of acculturative strategies for each nation, the Banting and Kymlicka (2004) multicultural classification was utilised identifying Australia as “strong” and Austria as “weak” in their multicultural policies, suggesting multicultural and monocultural (i.e., melting pot) acculturation strategies for these two countries, respectively. In addition to this classification, descriptive literature of the migratory experience of Bosnian refugees in these two countries was considered and is utilised to strengthen this classification. A short descriptive summary is presented in the next section.

## Resettlement of Bosnians in Australia and Austria

In the 1990s, Bosnians were the largest group of refugees who received humanitarian sponsored visas and were permanently resettled in Australia (Jupp, 2002). As such, they immediately received permanent residency and unrestricted access to all services afforded to Australian citizens including language training, access to health services, and income support. In addition, refugees resettled permanently in Australia had an option to reunite with their immediate family members and bring them to live in Australia (DIAC, 2009).

On the other hand, the huge influx of refugees and asylum applications in Austria and other western European countries in the 1990s had a major impact on the admission processes and the provision of protection under the 1951 Convention (United Nations, 1951). Instead of provision of humanitarian protection and permanent resettlement, Bosnian refugees who arrived in Austria were almost automatically provided with a temporary protection visa, were housed in temporary accommodations and were denied the right to work, receive social security benefits, or reunite with family members outside of Austria (Bauer, 2008; Franz, 2005). As a result, these *de facto* refugees found employment in the black market, enabling them to move out of crowded, temporary housing and eventually acquired working permits. Consequently, because of the newly introduced settlement quota system in the 1990s, Bosnians were granted residency permits only once they were employed or had an employed immediate family member (Krause & Liebig, 2011). Unfortunately, this process usually took years to achieve, exposing individuals and families to many years of stress associated with the migratory process. This illustrates the differences in the experiences in the post-migratory environments that these two groups of refuges faced upon leaving their war-torn countries.

## Aims of the current study

The current study proposes to investigate the impact of acculturative stress on mental health in Bosnian refugees who have experienced similar pre-migratory experiences associated with war exposure, but have settled in two different societies—Australia and Austria. Given that both of these refugee groups left Bosnia because of the war and have experienced traumatic events and losses associated with war and prosecution, it is important to understand the impact of these contextual factors in the acculturative process. Furthermore, the differences in resettlement policies would be expected to generate varying experiences of acculturative stress. Specifically, this study will investigate the relationships between war-related experiences, acculturative stress, and mental health in Bosnian refugees, and whether there are group differences in the above relationships between Bosnian refugees resettled in Austria and Australia. It was hypothesised that: (1) there will be no difference in levels of traumatic experiences reported by those refugees resettled in Australia and those resettled in Austria; (2) there will be no difference in levels of PTSD, and depressive and anxiety symptoms reported by refugees resettled in Australia and those resettled in Austria; (3) traumatic experience will be associated with increased levels of severity of PTSD and depressive and anxiety symptoms across both groups; and (4) increased acculturative stress would be associated with increased levels of severity of PTSD and depressive and anxiety symptoms more so in the sample of Austrian Bosnians than in the sample of Australian Bosnians.

## Method

### Participants

Participants were eligible for inclusion in this study if they were older than 18 years of age, resided in Australia or Austria during data collection, and were exposed to war events in Bosnia anytime between 1992 and 1995.

### Data collection

Participants in this study were recruited between January 2010 and January 2013 using various recruiting options including *snow-balling*; online recruitment; advertising in local media including newspapers, radio, and television; and recruitment through social clubs and groups whose members were of Bosnian background. Participants responded to an anonymous self-report survey via hosted website or a hard copy of the survey which was returned anonymously in a prepaid envelope. The majority of participants (*n*=78, 57%) utilised a hard copy version. There were no statistical differences between those who filled a hard copy or an online version of the survey for any of the outcomes including levels of PTSD (*χ* ^2^=31.54; *p*=.29), depressive (*χ* ^2^=17.86; *p*=.53), or anxiety symptoms (*χ* ^2^=12.26; *p*=.66).

### Measures

All measures used in the study were translated from English into Bosnian by the first author who is a native speaker and then back-translated by an independent bilingual academic allowing for comparison between the two translations. Any discrepancies in translation were resolved by discussion and mutual agreement between the first author and the bilingual academic.

### Sample characteristics

The questionnaire included a set of demographic questions (age, sex, education, and marital status). Trauma history questions (yes/no) were assessed in relation to war-related (e.g., torture, concentration camp, killing) and other traumatic events (e.g., exposure to disasters, accidents, and assaults) experienced during their lifetime.

### Posttraumatic Diagnostic Scale

The Bosnian translation (Powell & Rosner, 2005) of the *Posttraumatic Diagnostic Scale* (PDS; Part 3 only) (Foa, Cashman, Jaycox, & Perry, 1997) was used for the assessment of current PTSD symptomatology. This measure is based on the DSM-IV PTSD symptom criteria (APA, 1994), which included 17 items scored on a 4-point Likert scale (from 0=not at all or only one time, to 3=five or more times per week/very much/almost always). The alpha coefficient for the current Bosnian version of the PDS was excellent (α=.97).

### Depression and anxiety scale

The *Depression Anxiety Stress Scales* (DASS-21) is a 21-item self-report inventory designed to provide measures of the three related negative affective states of *depression, anxiety*, and *stress* (Lovibond & Lovibond, 1995). In the current study, only *depression* and *anxiety* scales were utilised and assessed for presence of symptoms over the past 2 weeks. Items are measured on a 4-point Likert scale (0=did not apply to me at all, and 3=applied to me very much, or most of the time). Alpha coefficients for the depression (α=.95) and anxiety (α=.92) scales in the current sample were excellent.

### Demands of immigration scale

The Demands of Immigration (DI) Scale (Aroian, Norris, Tran, & Schappler-Morris, 1998) was used to measure acculturative stress experienced over the last 6 months. This scale includes multiple subscales relating to *Loss* (longing for people, places and things in the homeland), *Novelty* (unfamiliarity with the tasks of daily living), *Occupation* (difficulty finding acceptable work), *Language* accommodation (having an inadequate level of English/German), *Discrimination* (perceived), and *Not feeling at home* (not feeling part of one's surrounding or social structure). Items are rated along a 6-point Likert scale (1=not at all to 6=very much). In the present study the Cronbach's alpha for the total DI scale was excellent (α=.94) and ranged between .75 and .87 for the subscales, indicating good internal consistency.

### Statistical analyses

Differences in demographics, mental health, and subscales of the DI between the refugees resettled in Australia and Austria were examined using *t*-tests and chi-square analyses. Hierarchical multiple regressions were used (controlling for exposure to traumas and demographic variables) to examine the effects of acculturative stress on PTSD, depression, and anxiety symptoms in the subgroups of refugees resettled in Australia and Austria. All data were analysed in SPSS version 22. This study was reviewed and approved by the Monash University Human Research Ethics Committee, certificate number CF09/3238–2009001758.

## Results

### Demographics

The demographic characteristics of the sample are presented in Table 1. In total, 138 participants were recruited into the study with 55% being male with a mean age of 40.20 years (range between 18–80 years). Forty-one percent of participants resided in Australia and 59% resided in Austria, with those living in Australia being significantly older. Refugees who resettled in Austria had a significantly longer length of residence than those who resettled in Australia.

**Table 1:** Socio-demographic characteristics of participants

	Australian Bosnians *N*=56 M/n (SD/%)	Austrian Bosnians *N*=82 M/n (SD/%)	Group differences
Age	44.61 (14.60)	37.20 (14.44)	*t*(136)=2.95, *p*=.004
Sex			*χ* ^2^=2.85, *p*=.065
Male	26 (46.4%)	50 (61%)	
Marital status			*χ* ^2^=1.18, *p*=.186
Married/in a relationship	43 (76.8%)	56 (68.2%)	
Single	13 (23.2%)	26 (31.7%)	
Education level			*χ* ^2^=3.36, *p*=.645
Postgraduate	6 (10.7%)	12 (14.6%)	
Tertiary	18 (31.1%)	21 (25.6%)	
Advanced Diploma	9 (16.1%)	19 (23.2%)	
High school	16 (28.6%)	24 (29.3%)	
Secondary or less	6 (10.7%)	4 (4.9%)	
Other	1 (1.8%)	2 (2.4%)	
Length of residence (in years)	16.05 (3.46)	18.76 (5.69)	*t*(136)=−3.17, *p*=.002

### Exposure to traumatic events

Eighty-two percent of the whole sample reported experiencing at least one traumatic event, while 70% reported experiencing three or more traumatic events in their life (M=5.09, SD=4.03, range 0–16). Similarly, 80% of the whole sample reported experiencing at least one war-related event, and 64% reported experiencing more than three war-related traumatic events (M=3.84, SD=2.95, range 0–11 events). Table 2 lists the types of traumatic events experienced by Bosnian refugees living in Australia and Austria. The most common experiences reported by participants include experiencing separation from family, direct bombardment, and sniper fire. There were no differences in traumatic exposure reported by men and women, but there were significant differences based on the country of resettlement, with Australian Bosnians reporting more traumatic exposure M=6.68 (4.00) than Austrian Bosnians M=4.01 (3.70); *t*(136)=4.02, *p*<.001.

**Table 2:** Traumatic events reported by Bosnian refugees living in Australia and Austria

Traumatic experience	Australian Bosnians *N*=56 *n* (%)	Austrian Bosnians *N*=82 *n* (%)	Group differences
Separation from family	41 (73%)	35 (43%)	*χ* ^2^=12.30, *p*=.001
Direct bombardment or sniper fire	34 (60%)	35 (43%)	*χ* ^2^=3.60, *p*=.064
Other stressful event happened to family	30 (54%)	26 (32%)	*χ* ^2^=5.30, *p*=.030
Family member injured, killed, or tortured	30 (54%)	25 (30%)	*χ* ^2^=7.25, *p*=.009
Other stressful or upsetting event	25 (45%)	27 (33%)	*χ* ^2^=1.45, *p*=.279
Life-threatening accidents	20 (36%)	21 (26%)	*χ* ^2^=1.23, *p*=.340
Serious physical attack or assault	16 (29%)	18 (22%)	*χ* ^2^=.54, *p*=.546
Witnessed family injury, killing, or torture	10 (18%)	5 (6%)	*χ* ^2^=4.30, *p*=.050
Fire, flood, or natural disaster	9 (16%)	5 (6%)	*χ* ^2^=3.25, *p*=.088
Combat	8 (14%)	2 (3%)	*χ* ^2^=6.77, *p*=.016
Torture	7 (13%)	4 (5%)	*χ* ^2^=1.52, *p*=.232
Concentration camp	5 (9%)	4 (5%)	*χ* ^2^=.72, *p*=.489
War-related serious injury	4 (7%)	0 (0%)	*χ* ^2^=7.13, *p*=.029

Note: *χ*^2^=Fisher's exact test.

### Mental health outcomes

As can be seen in Table 3, Australian Bosnians reported significantly higher levels of PTSD, depressive, and anxiety symptoms compared to Austrian Bosnians.

**Table 3:** Means, standard deviations and univariate analyses for mental health symptoms and acculturative stress reported by refugee group

	Australian Bosnians *N*=56	Austrian Bosnians *N*=82	
	M (SD)	M (SD)	Group differences
PDS	13.29 (14.28)	5.37 (7.93)	*t*(71.06)=3.60, *p*=.001
DASS-depression	5.42 (5.80)	2.69 (4.36)	*t*(83.99)=2.85, *p*=.006
DASS-anxiety	4.94 (5.38)	2.42 (3.58)	*t*(76.77)=2.92, *p*=.005
*Demands of Immigration Subscales*			
Loss	10.12 (3.52)	9.41 (3.35)	*t*(130)=1.15, *p*=.251
Language	7.53 (3.56)	5.50 (3.23)	*t*(129)=3.37, *p*=.001
Not at home	8.10 (3.40)	7.85 (3.30)	*t*(130)=.385, *p*=.701
Novelty	10.46 (4.31)	8.78 (4.32)	
Occupation	11.84 (5.94)	11.61 (5.98)	*t*(129)=.216, *p*=.830
Discrimination	9.41 (4.44)	10.48 (3.94)	*t*(128)=−1.43, *p*=.153
Total DI	52.13 (18.75)	48.80 (18.17)	*t*(129)=1.01, *p*=.315

Note: PDS=Posttraumatic Diagnostic scale; DASS-Depression=Depression, Anxiety, and Stress scale; DI=Demands of Immigration scale.

### Acculturative stress

Except for the subscale *Language*, levels of acculturative stress did not differ significantly between Australian and Austrian Bosnians. As indicated in Table 3, Australian Bosnians reported significantly more stress associated with accommodating to the host language compared to Austrian Bosnians.

### Predictors of mental health outcomes

Hierarchical regression analyses were conducted to examine significant predictors of PTSD, depressive, and anxiety symptoms separately for the Austrian and Australian samples, while controlling for exposure to traumatic events and demographic variables. As can be seen in Tables 4, 5 and 6, there were differences in the number and type of predictors for the two samples for PTSD, depressive, and anxiety symptoms. In particular, when controlling for age, sex, and traumatic exposure (Table 4), acculturative stress contributed significantly to explain 57% of variance in PTSD symptoms *F*(9,119)=18.49, *p*<.001. When models were tested separately for each refugee sample, the model was only significant for the Austrian Bosnian group. In particular, only the subscales of *Loss* contributed significantly to explain 58% of the variance in the PTSD symptoms *F*(9,73)=12.26, *p*<.001.

**Table 4:** Hierarchical regression analyses for PTSD symptoms scores reported by refugee group

	Total sample (*N*=119)			Australian Bosnians (*N*=46)			Austrian Bosnians (*N*=73)		
			
	Model 1	Model 2	Model 3	Model 1	Model 2	Model 3	Model 1	Model 2	Model 3
Adjusted R^2^	.211***	.528***	.570*	.169**	.468***	.488	.145**	.533***	.581*
Age	.444***	.276***	.151∧	.473**	.336**	.216	.333**	.220**	.143
Female sex	.169*	.133*	.125*	.196	.124	.198	.207∧	.219**	.163*
Traumatic events		.588***	.558***		.562***	.424**		.630***	.604***
DI-Loss			.155*			.107			.230*
DI-Language			.059			−.013			.010
DI-Not at home			.283*			.285			.225∧
DI-Novelty			−.089			.124			−.235
DI-Occupation			−.029			−.072			.184
DI-Discrimination			−.130			−.018			−.229∧

Note: DI=Demands of Immigration scale;

* *p*<.05,

** *p*<.01,

*** *p*<.001;

∧ *p*<.10.

**Table 5:** Hierarchical regression analyses for depressive symptoms scores reported by refugee group

	Total sample (*N*=119)			Australian Bosnians (*N*=46)			Austrian Bosnians (*N*=73)		
			
	Model 1	Model 2	Model 3	Model 1	Model 2	Model 3	Model 1	Model 2	Model 3
Adjusted R^2^	.071**	.373***	.375	.067	.372***	.365	.010	290***	.331
Age	.283**	.123	.048	.351*	.191	.206	.114	.023	−.054
Female sex	.091	.045	.049	.066	−.019	.071	.141	.142	.138
Traumatic events		.574***	.583***		.570***	.444**		.538***	.597***
DI-Loss			.122			.090			.155
DI-Language			.075			−.188			.331∧
DI-Not at home			.019			.031			−.058
DI-Novelty			−.074			.307			−.454*
DI-Occupation			−.086			−.147			.008
DI-Discrimination			.122			.226			.156

Note: DI=Demands of Immigration scale;

* *p*<.05,

** *p*<.01,

*** *p*<.001;

∧ *p*<.10.

**Table 6:** Hierarchical regression analyses for anxiety symptoms scores reported by refugee group

	Total sample (*N*=119)			Australian Bosnians (*N*=46)			Austrian Bosnians (*N*=73)		
			
	Model 1	Model 2	Model 3	Model 1	Model 2	Model 3	Model 1	Model 2	Model 3
Adjusted R^2^	.094**	.446***	.466	.087∧	.382***	.411	.022	.434***	.493*
Age	.322***	.149*	.001	.382*	.231	.201∧	.160	.051	−.123
Female sex	.086	.037	.062	.081	−.003	.135	.132	.133	.158∧
Traumatic events		.618***	.571		.562***	.356*		.649***	.678***
DI-Loss			.087			.083			.093
DI-Language			.175			−.107			.489**
DI-Not at home			.116			.208			−.022
DI-Novelty			−.043			.312			−.357*
DI-Occupation			−.024			−.091			.059
DI-Discrimination			−.004			.060			.000

Note: DI=Demands of Immigration scale;

* *p*<.05,

** *p*<.01,

*** *p*<.001;

∧ *p*<.10.

Table 5 shows the hierarchical regression analysis for depressive symptoms scores. Acculturative stress was not a significant predictor of depressive symptoms for the total sample or when considered separately for each refugee sample. The number of traumatic events was the only significant predictor of depressive symptoms.

Similarly, acculturative stress was not a significant predictor of anxiety symptoms in the total sample or in the Australian Bosnian group (Table 6). However, after controlling for age, sex, and traumatic exposure, the acculturative stress subscales of *Language* and *Novelty* contributed significantly to explain 49% of the variance in anxiety symptoms *F*(9,74)=9.01, *p*<.001 for the Austrian Bosnian group.

## Discussion

This study investigated the level of traumatic exposure and prevalence of mental health symptoms in two samples of Bosnian refugees resettled in Australia and Austria. Furthermore, mental health impact of age, sex, exposure to traumatic events and acculturative stress was investigated across both samples.

The results show high levels of exposure to traumatic events and high prevalence rates of PTSD, depressive, and anxiety symptoms across both samples, confirming prior research conducted with refugees in general (Fazel et al., 2005; Silove, Steel, McGorry, & Mohan, 1998) and other Bosnian samples around the world (Knipscheer & Kleber, 2006; Mollica et al., 2001; Momartin, Silove, Manicavasagar, & Steel, 2003). However, contrary to our hypothesis, significant differences were also visible between the two groups included in this study. In particular, the Australian Bosnians were found to have experienced significantly more traumatic events and reported more PTSD, depressive, and anxiety symptoms than Bosnians resettled in Austria. Australian Bosnians were significantly older than Austrian Bosnians, which has been associated with more mental health symptoms in refugees (Bogic et al., 2012; Porter et al., 2005), and might explain the significantly more traumatic exposure and severity of posttraumatic symptoms identified in Australian Bosnians. In addition, this difference may be explained by different resettlement processes. In particular, Bosnian refugees that resettled in Australia went through resettlement under the United Nations High Commissioner for Refugees scheme, which granted resettlement and residence to the most vulnerable and to those who experienced particularly traumatic ordeals during the war (United Nations High Commissioner for Refugees, 2011). This is furthermore indicated by significant differences in types of traumatic events experienced and subsequently rationalises the higher rates of mental health symptoms found in the Australian Bosnians. These results also highlight the diversity of traumatic exposure found within the same refugee population, and are consistent with prior research conducted with ex-Yugoslav refugees, including Bosnians resettled in European countries that identified varying degrees of exposure and posttraumatic symptoms across samples resettled in different countries (Bogic et al., 2012).

Results also indicated that prior exposure to traumatic events remained the most powerful predictor of PTSD symptoms, which is consistent with previous research conducted with refugees (Bogic et al., 2012; Fazel et al., 2005; Ozer et al., 2008; Steel et al., 2002, 2009).

As predicted, accuturative stress was significantly associated with mental health. Particularly, in addition to exposure to traumatic events, acculturative stress was associated with greater experiences of cultural loss and nostalgia, contributing to more severe PTSD symptoms in the Austrian Bosnian group. In addition, the results suggested that Austrian Bosnians who experienced more language difficulties and were less occupied with novel tasks of daily living were more likely to report anxiety symptoms. These results are in agreement with prior research (Schweitzer et al., 2011; Söndergaard & Theorell, 2004) and indicate that some domains of acculturative stress promote mental health while others hinder it (Ellis et al., 2008, 2010; Knipscheer & Kleber, 2006). They suggest that bicultural (i.e., ethnic and host) orientation benefits mental health, as the ability to effectively negotiate between culturally appropriate behaviours enables refugees to learn the necessary skills to function in the host society (e.g., acquire host language), while still holding onto their cultural aspects that promote better mental health.

However, as these results indicate, this may not be possible for all refugees, as the impact of the acculturative stressors differed across the two samples of refugees of the same cultural background that resettled in different host countries examined in this study. This difference suggests that the acculturative context and conditions of the host society may impose different acculturative stressors for refugees. As proposed by Berry's acculturation model (1997, 2006), the host society can impact on the acculturation process of refugees by imposing either encouraging or less desirable acculturative strategies that consequently either encourage or oppose ethnic diversity and participation in the larger society. As described in the current study, Austrian Bosnians generally experienced temporary residence with limited benefits and rights, while their counterparts in Australia experienced a supportive migration context, as they immediately received permanent residency, language training, access to health services, and income support. Such a supportive approach to resettlement may have left refugees free to engage and practice their own culture and/or engage with the host culture, which may have removed the additional cumulative impact of acculturative stressors identified in the Austrian Bosnian sample. Besides, establishing secure residence has been found to be a stabilizing factor in the recovery process from trauma-related symptoms (Silove et al., 2007). While these findings confirm previous research, no causal pathway could be explored in this study, and further research is needed to fully explore these relationships.

Furthermore, and contradictory to our hypothesis, acculturative stress was not found to be a significant predictor of depressive symptoms as exposure to traumatic events was the only significant predictor of depressive symptoms across the two refugee groups in this study. These findings might be explained by considering the duration of resettlement. In particular, prior research in this area indicated that depressive symptoms associated with migration increase during the initial period of resettlement and then begin to decrease with increased length of stay in the host country (Fenta et al., 2004; Tran, Manalo, & Nguyen, 2007). These results seem to be consistent with the current sample of refugees who have been resettled in their respective host societies for a mean of 16 and 18 years (Australian and Austrian groups respectively), indicating a diminishing impact of the acculturative stressors in the later stages of resettlement.

### Implications

Resettled Bosnian refugees report high levels of trauma-related mental health problems many years after the war (>16 years) indicating the continuing need for support from health and social services (Bogic et al., 2012). Building on our understanding of the trauma-related risk factors and challenges facing refugees, these findings indicate that health and social services supporting refugees should not be limited to evaluations of psychopathological risk factors only. The contributing effect of acculturative stress and the cultural and social stressors experienced in resettlement needs to be considered as well, particularly as they offer a potential target for intervention. While prior research and guidelines have been developed to draw attention to migratory stressors in the conflict zones, refugee camps, and early in the resettlement (Miller & Rasmussen, 2010), the findings of the current study indicate that more could be done to address the long-term effects of traumatic exposure and migratory stress in assisting refugees’ adaptation to the new environment in the long term. Particularly, attention should be paid to enabling the refugees to maintain cultural and traditional aspects of their culture, as enabling them to do so may alleviate some of the migratory stressors associated with participation and functioning in the host society. Governmental immigration and resettlement policies that aim to promote successful adaptation of refugees should target psycho-socio-cultural stressors impacting mental health. Indeed, the adaptation of refugees may be best supported by policies that value cultural variety and inclusion and promote multiculturalism.

Furthermore, models that incorporate both trauma-focused interventions and support sociocultural adaptations of refugees should be promoted and integrated at the community level. The advantage of such models (e.g., Miller & Rasmussen, 2010; Silove, 1999) is that they target trauma and acculturative processes on an individual but also on a collective community level, therefore not only supporting the psychological well-being of individuals, but also assisting ethnic groups’ adaptation into the wider host society.

### Limitations

There are several methodological limitations in this study. The first refers to the difficulties inherent in conducting research with refugees (Jacobsen & Landau, 2003). The current study included a relatively small convenience sample of refugees recruited using the snowballing method and thus the generalisability of the results is limited. In addition, retrospective reporting and reliance on self-reporting may run a risk of not remembering or misrepresenting the events and non-accurate measurement of symptoms (Kessler, Wittchen, Abelson, Zhao, & Stone, 2000), presenting a risk of recall bias (Southwick, Morgan, Nicolaou, & Charney, 1997). Nonetheless, this study has contributed to the literature by providing information about resettlement experiences of Bosnian refugees who resettled in Australia and Austria and their impact on mental health. Another limitation is the possibility of selection bias rather than real differences between the refugee groups. The sample of refugees in this study may be more open to reporting their distress than the refugees in the host communities targeted who did not take part in the study. Finally, the DI scale has not been validated in the Bosnian refugee population.

## Conclusion

The results of this study indicate that, in addition to pre-migratory traumatic exposure, acculturative stress has an effect on presentation of PTSD and anxiety, but not depressive symptoms. However, this effect was only found for some domains of acculturative stress indicating that some post-migratory stressors hinder the recovery from traumatic exposure while others may support recovery. Furthermore, this study offered a direct comparison of Bosnian refugees living in two different host societies. The findings indicated that the relationship between acculturative stress and mental health outcomes varied according to the country of resettlement, suggesting that acculturative stress experienced by Bosnian refugees may be influenced by the social context of the host society, in particular the immigration policies and attitudes of the wider society toward different cultures. In summary, findings of this study and group differences identified suggest that mental health of refugees is influenced by refugees’ characteristics (e.g., traumatic exposure), post-migration risk factors (e.g., acculturative stressors), and resettlement trajectories (e.g., host nations’ policies and attitudes). It should be pointed out that no causal pathway can be established with these results and further research is needed to replicate these findings in larger samples of refugees of different cultural backgrounds living in different host nations.
